# Supplementary material for: Efficacy and safety of guide extension catheter in balloon pulmonary angioplasty for treatment of complex lesions in chronic thromboembolic pulmonary hypertension
Source: PLoS One. 2023 Jan 27;18(1):e0280683. doi: 10.1371/journal.pone.0280683 (PMC9882884; doi:10.1371/journal.pone.0280683)
Supplement: Attachment — Submitted filename: PONE-D-22-27862review.docx [file pone.0280683.s003.docx]

In this study, the author retrospectively analyzed 91 lesions in 28 patients with chronic thromboembolic pulmonary hypertension who underwent balloon pulmonary angioplasty using a guide extension catheter. The procedure of catheter in balloon pulmonary angioplasty was effective and safety.

It might be better to compare the efficacy and safety of conventional catheterization and catheter in balloon pulmonary angioplasty.
